# Supplementary material for: Optimal Efficacy and Safety of Humanized Anti-Scg3 Antibody to Alleviate Oxygen-Induced Retinopathy
Source: Int J Mol Sci. 2021 Dec 29;23(1):350. doi: 10.3390/ijms23010350 (PMC8745183; doi:10.3390/ijms23010350)
Supplement: Supplementary file 1 [file ijms-23-00350-s001.zip › ijms-1411886-supplementary.pdf]

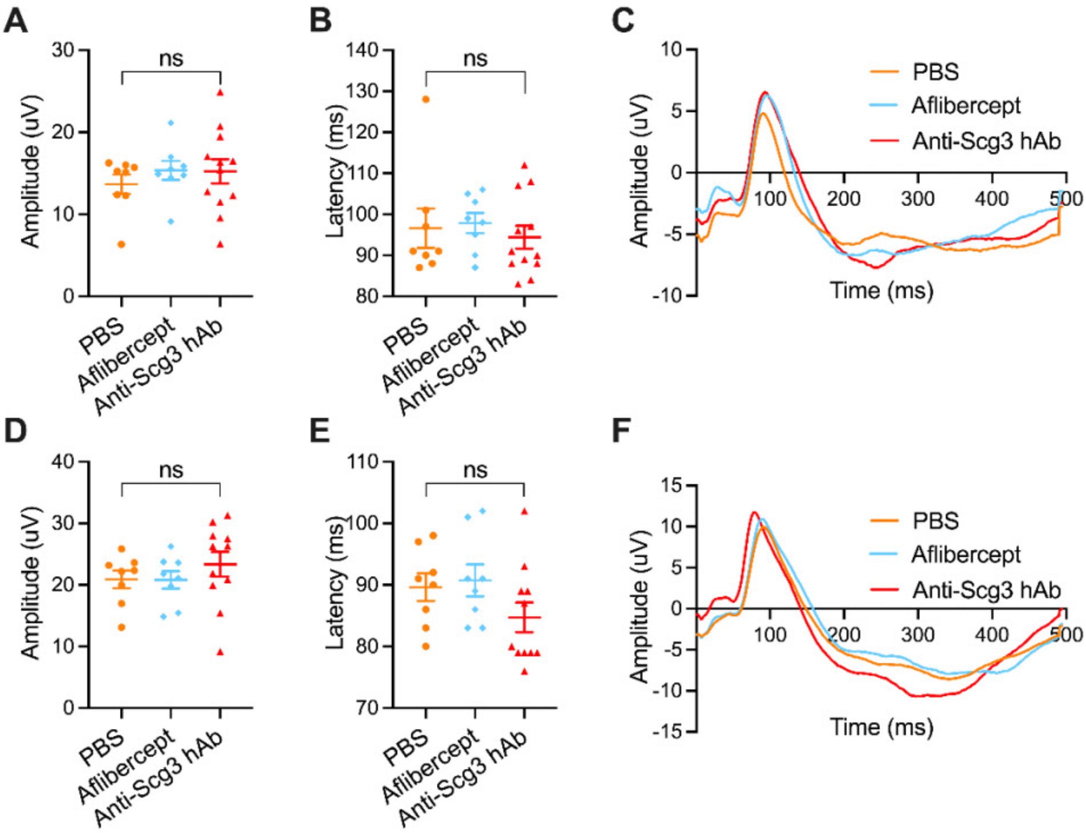

Figure S1. Pattern electroretinogram (PERG) to assess the function of retinal ganglion cells in the inner retina of OIR mice. (A) PERG amplitude at P21 (7 DPI). (B) PERG latency at P21. (C) Average ERG graphs of all mice in each group at P21. n = 8 eyes (PBS), 8 eyes (aflibercept) and 12 eyes (anti-Scg3 hAb). (D) PERG amplitude at P42. (E) PERG latency at P42. (F) Average ERG graphs of all mice in each group at P42. n = 8 eyes (PBS), 8 eyes (aflibercept) and 11 eyes (anti-Scg3 hAb). No difference was detected among anti-Scg3 hAb, aflibercept and PBS via intravitreal injection. ± SEM. ns, not significant; one-way ANOVA test.

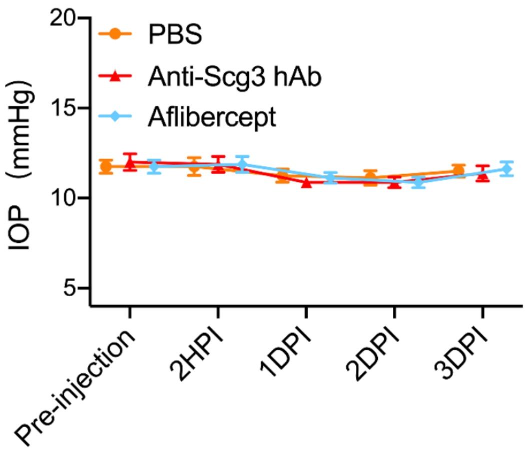

Figure S2. Intraocular pressure (IOP) remains stable after anti-Scg3 hAb or aflibercept treatment. IOP was monitored for OIR mice treated with anti-Scg3 hAb, aflibercept or PBS through intravitreal injection at P14. No significant change was detected in IOP up to 3 DPI. ± SEM. n = 8 eyes (PBS), 8 eyes (aflibercept) and 8 eyes (anti-Scg3 hAb); one-way ANOVA test.

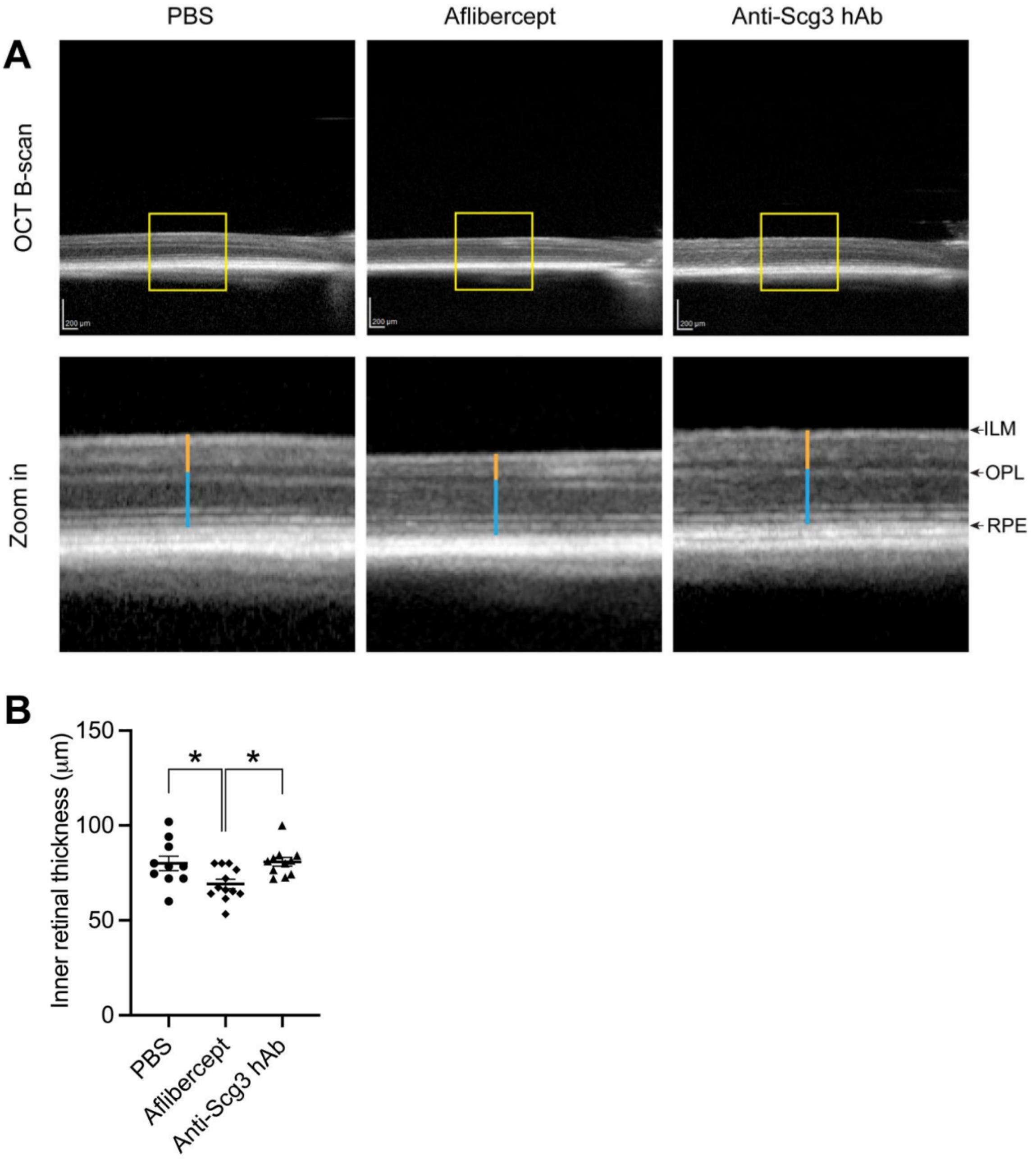

Figure S3. Intravitreal aflibercept decreases the inner retinal thickness in OIR mice at P42. (A) Representative OCT B-scan of the retinal thickness at P42. Yellow box corresponds to the magnified area below, showing retinal structure. PBS, aflibercept (2 µg/0.5 µl/eye), or anti-Scg3 hAb (2 µg/0.5 µl/eye) was injected intravitreally with the same agents for both eyes of OIR mice at P14. OCT B-scan was performed at P42. Inner retinal layer was defined from inner limiting membrane (ILM) to the surface of outer plexiform layer (OPL). Orange line represents inner retinal thickness. Outer retinal layer was defined from OPL to the surface of retinal pigment epithelium (RPE). Blue line represents outer retinal thickness. (B) Quantification of inner retinal thickness in A. n = 10 (PBS), 12 (aflibercept) and 11 eyes (anti-Scg3 hAb). ±SEM; * $P<0.05$; one-way ANOVA test.
